# Radiofrequency Chondroplasty May Not Have a Long-Lasting Effect in the Treatment of Concomitant Grade II Patellar Cartilage Defects in Humans

**DOI:** 10.3390/jcm9041202

**Published:** 2020-04-22

**Authors:** Ulrich Koller, Bernhard Springer, Colleen Rentenberger, Pavol Szomolanyi, Wenzel Waldstein, Reinhard Windhager, Siegfried Trattnig, Sebastian Apprich

**Affiliations:** 1 Department of Orthopedics and Trauma Surgery, Medical University of Vienna, Waehringer Guertel 18–20, 1090 Vienna, Austria; ulrich.koller@meduniwien.ac.at (U.K.); bernhard.springer@meduniwien.ac.at (B.S.); colleen.rentenberger@meduniwien.ac.at (C.R.); reinhard.windhager@meduniwien.ac.at (R.W.); sebastian.apprich@meduniwien.ac.at (S.A.); 2 High-Field MR Centre, Department of Biomedical Imaging and Image-guided Therapy, Medical University of Vienna, Waehringer Gürtel 18–20, 1090 Vienna, Austria; pavol.szomolanyi@meduniwien.ac.at (P.S.); siegfried.trattnig@meduniwien.ac.at (S.T.); 3 Institute of Measurement Science, Slovak Academy of Sciences, Dúbravská cesta 5801/9, 84104 Karlova Ves, Bratislava, Slovakia; 4 CD Laboratory for Clinical Molecular MR Imaging, Medical University of Vienna, Waehringer Gürtel 18-20, 1090 Vienna, Austria; 5 Austrian Cluster for Tissue Regeneration, Donaueschingenstr 13, 1200 Vienna, Austria

**Keywords:** patellar cartilage defect, arthroscopy, radiofrequency chondroplasty, T2 mapping, MRI

## Abstract

The effect of radiofrequency chondroplasty on cartilage tissue is not well studied. This prospective pilot study investigates the effect of radiofrequency chondroplasty on International Cartilage Repair Society (ICRS) grade II patellar cartilage defects using high-resolution magnetic resonance imaging (MRI) with T2 mapping. Six consecutive patients were treated for ICRS grade II patellar cartilage defects using radiofrequency chondroplasty. Before surgery and at defined follow-ups (2 weeks, 4 and 12 months) a high-resolution morphological 3 Tesla MRI with quantitative T2 mapping was performed. At baseline MRI, global T2 values of cartilage defects were increased (46.8 ms ± 9.7) compared to healthy cartilage (35.2 ms ± 4.5) in the same knee which served as reference. Two weeks after treatment, global T2 values (39.2 ms ± 7.7) of the defect areas decreased. However, global T2 values of the defect areas increased beyond the preoperative levels at 4 months (47.4 ms ± 3.1) and 12 months (51.5 ms ± 5.9), respectively. Zonal T2 mapping revealed that the predominant changes in T2 values occurred at the superficial cartilage layer. T2 mapping appears to be an ideal method to monitor cartilage degeneration after chondroplasty. Based on the small sample size of this pilot study, radiofrequency chondroplasty may cause cartilage damage and may not have a long-lasting effect in the treatment of grade II patellar cartilage defects. In five out of six patients, postoperative cartilage damage was observed on quantitative MRI. This study was therefore terminated before completion. We recommend only addressing the pathology which indicated arthroscopy and leaving concomitant cartilage lesions untreated.

## 1. Introduction

Chondral lesions are a common additional finding occurring in up to 61% of all knee arthroscopies [1,2,3]. Cartilage lesions are graded according to the International Cartilage Repair Society (ICRS) classification system [4,5]. Whereas clear recommendations for the treatment of ICRS grade IV lesion exist [6], surgical treatment of early cartilage lesions (ICRS grade 1–2) remains controversial.

In addition to mechanical debridement and shaving, thermal application through monopolar or bipolar radiofrequency may be used for the treatment of grade II lesions [7,8,9]. Two effects are created by exposing the cartilage tissue to heat. First, a so-called neo-surface is created by uncoiling the collagen triple helix, which is reformed in a less organized manner after cooling down [10]. This neo-surface is thought to be more resistant against shear stress, which might slow down the degradative process. The second effect is an annealing effect that makes the surface less permeable for water. As a result, water remains within cartilage tissue [11,12,13,14]. On the other side, potential side effects of radiofrequency chondroplasty have been reported. These include osteonecrosis [15], cell death due to temperatures over 50 °C [16] and progression of the cartilage lesion [14,17].

The surgical success of radiofrequency chondroplasty has so far only been measured by clinical outcome scores and radiographs [7,12,18]. However, the effect of this procedure on the cartilage tissue itself is not well investigated. The current gold standard for non-invasive visualization of the hyaline cartilage in vivo is magnetic resonance imaging (MRI) [19,20,21]. In addition to the standard morphological MRI, quantitative biochemical MRI has evolved [19,22].

The quantitative T2 mapping technique [23] represents a sensitive MR technique that provides information on the interaction between the extracellular matrix and water molecules of the cartilage. Healthy cartilage shows increasing T2 values from the deep to the superficial zone of the cartilage [24,25,26,27,28,29]. It has been shown that increased T2 values indicate damage in the collagen construct, reflecting the increased water mobility [25,30,31,32]. T2 mapping is, therefore, an ideal marker to detect and monitor progression of osteoarthritis [33].

The purpose of the present prospective study was to evaluate the longitudinal outcome of patients with ICRS grade II patellar cartilage defects treated with radiofrequency chondroplasty by means of high-resolution morphological and quantitative MRI over the time period of 1 year. The hypothesis was that radiofrequency chondroplasty leads to an improvement in MRI values.

## 2. Materials and Methods

All subjects gave their informed consent for inclusion before they participated in this study. This study was conducted in accordance with the Declaration of Helsinki of 1975, revised in 2013, and the protocol was approved by the Ethics Committee of the Medical University of Vienna (EK 1184/2016). Initially, 20 patients were intended for this study. This study was planned as a pilot study. For this reason, no power analysis was performed. However, due to reasons stated below, this study was terminated after the inclusion of the sixth patient.

Inclusion criteria were defined as follows: indication for arthroscopic surgery for medial meniscus tear [34] and concomitant presentation of ICRS grade II cartilage defect at the patella. Inclusion eligibility was determined preoperatively on MRI and confirmed by diagnostic arthroscopy. Only patients with grade II patellar cartilage defects were included. Exclusion criteria were defined as follows: history of patellar dislocation, patellar fracture or previous surgeries on the affected knee, ligamentous instability, cartilage defects in the medial and lateral femorotibial compartment, age >50 years, BMI >31 kg/m^2^, or any contraindications for MRI examination. None of the patients reported anterior knee pain or pain walking downwards associated with a patellofemoral pathology.

The Visual Analog Scale, the Knee Injury and Osteoarthritis Outcome Score (KOOS) and the UCLA Activity Score were used before surgery and at last follow-up.

**Figure 1:**
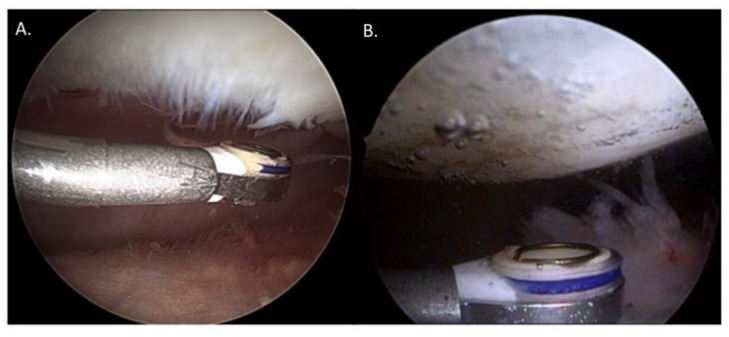
(**A**) Intraoperative finding of the articular cartilage grade II lesion on the medial patella and (**B**) directly after radiofrequency chondroplasty, respectively.

All interventions and the intraoperative grading of cartilage defects were performed by an experienced consultant orthopedic surgeon. Standard arthroscopy was performed utilizing anterolateral and anteromedial portals. After documentation of the size and location of the patellar cartilage defect, treatment with the Paragon T2 Wand (ArthroCare, Smith&Nephew) instrument was performed. The defect was sealed, by painting the probe over the degenerated cartilage surface. This was done in a standardized manner until a “neo-surface” was formed (Figure 1). The probe has a temperature indicator to prevent cartilage from being damaged by heat. This indicator turns blue when the heat exceeds 50 °C. In all six cases, trimming of the medial meniscus was performed. After arthroscopy, a drain was routinely inserted and removed on the first postoperative day. Patients were mobilized with crutches for 10–14 days on full weight bearing and full range of motion without any brace. No signs of infection or unusual swelling occurred in any patient.

The MRI protocol was identical for all patients and time points of follow up. MRI was performed on a 3.0 T whole-body Magnetom TimTrio scanner (Siemens Medical Solutions, Erlangen, Germany), using a gradient strength of 40 mT/m and an eight-channel knee array coil (IN vivo, Gainesville, FL, USA). Patients were positioned in the supine position, with the extended knee tightly fixed and the joint space in the middle of the coil.

For the morphological evaluation, the MRI protocol consisted of a high-resolution, proton-density (PD) turbo-spin-echo (TSE) sequence which was obtained for the patella in the axial plane (TR 2220 ms; TE: 38 ms; FoV 120 mm × 120 mm; acquisition matrix size 448 × 403; recalculated matrix size 896 × 896, voxel size 0.13 mm × 0.13 mm × 2 mm; 25 slices; interslice gap 10%; flip angle 180°; bandwidth 180 Hz/pixel; scan time 2:53 min). The pixel size of 200 µm for the morphological proton density (PD) turbo-spin-echo (TSE) sequence remained below the level that is deemed required to reliably show superficial alterations of articular cartilage [35].

An axial multi-echo spin-echo (MESE) T2 sequence for T2 mapping (repetition time (TR) 1440 ms; 8 echo times (TE) 11.9–95.2 ms; field of view (FoV) 140 mm × 140 mm; pixel matrix 320 × 320; voxel size 0.43 mm × 0.43 mm × 3 mm; bandwidth 200 Hz/pixel; 14 slices; scan time 4:09 min) was also performed [36]. This sequence was put at the end of the MR protocol to avoid any bias from loading effects to the T2 values.

Morphological and T2 mapping sequences were planned on the same set of localizers. Acquired images were oriented perpendicular to the patella surface in the axial plane.

Any additional sequences performed during the scans were not used for this analysis.

The cartilage surface of the patella was morphologically graded based on a modified ICRS classification system as already mentioned in the introduction [4].

T2 relaxation times were derived from on-line reconstructed T2 maps using a pixel-wise, mono-exponential, non-negative least squares (NNLS) fit analysis (MapIt, Siemens Medical Solutions, Erlangen, Germany). To verify the localization and size of cartilage defects, we used the 3D spatial presentation from the high-resolution true fast imaging with steady-state free precession (FISP) images. With the help of header information and anatomical landmarks, the defects were reproduced on the T2 maps using the second echo images (TE = 13.8 ms), as these have the best signal-to-noise-ratio (SNR). T2 values from the subchondral bone to the cartilage surface (global T2) were assessed on manually drawn regions of interest (ROIs) on one to three consecutive slices of the T2 map, depending on defect size. In addition, a reference area with a morphologically normal appearance on PD TSE images, i.e., with preserved cartilage thickness, intact surface, and without intrachondral signal alterations, was identified in the same patient, either on the contralateral facet of the patella or at least three slices away from the defect in the cranio-caudal direction. Corresponding ROIs for the assessment of the T2 values were manually drawn on one to three slices similar in size to the ROI and these served as a reference.

Subsequently, to evaluate the impact of the radiofrequency chondroplasty on different cartilage layers, zonal T2 values were evaluated by dividing the global ROIs into two layers, a deep (deep T2) and a superficial one (superficial T2). For this purpose, the full cartilage layer thickness was separated into two ROIs: the deep layer reaches from the subchondral lamina up to one half of cartilage thickness and the superficial layer includes the remaining cartilage tissue towards the intraarticular surface. Adjustments for ROIs were made for localization and in cases of cartilage loss over time. Special attention was paid so as not to include any free synovial fluid, partial volume effects, or artifacts.

Previous studies have demonstrated a high intra- and interrater reliability for the assessment of T2 values [37,38].

The acquisition and analysis of all MRIs was performed in a specialized center with long-standing experience in MRI-related research.

Statistical evaluation was performed using SPSS (version 23.0, SPSS institute, Chicago, IL, USA) for Mac. Descriptive evaluation used mean values, standard deviations and 95% confidence levels. Differences in T2 values between different time points were evaluated using one-way ANOVA with posthoc Duncan test. *P* values < 0.05 were assumed as statistically significant.

## 3. Results

This study included four male patients and two female patients, with a median age of 48 years (range 41–48) and a median BMI of 28.3 kg/m^2^ (range 20.4–30.7).

### 3.1. Clinical Scoring

The mean KOOS increased from 113 ± 22.8 to 156 ± 10.6 points (*p* = 0.004). The mean UCLA Activity Score increased from 6 ± 1.2 to 9 ± 0.8 points (*p* = 0.002). A significant decrease in pain was observed from a mean preoperative Visual Analogue Scale (VAS) score of 5 ± 2.2 to 1 ± 0.9 at one year (*p* = 0.015).

### 3.2. Global T2 Values

**Figure 2:**
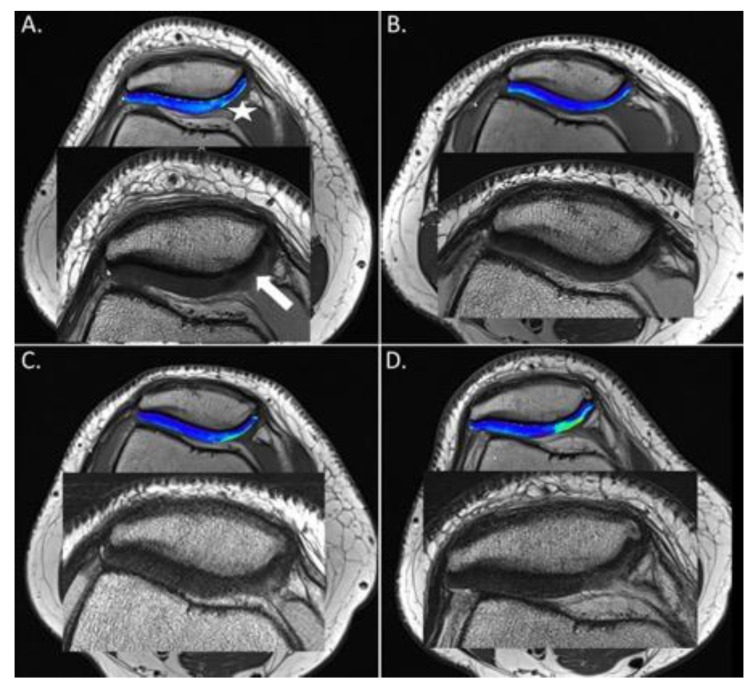
(**A**). Preoperative morphological magnetic resonance imaging (MRI) showing the International Cartilage Repair Society (ICRS) grade 2 cartilage defect at the medial facet of the patella (white arrow) and above the corresponding, color-coded T2 map. Note the increased T2 relaxation times of the defect, especially at the transition from the apex to the medial facet. (white star). (**B**) Two weeks postoperatively, the morphological MRI shows a full restoration of the cartilage surface including a hypointense area. The corresponding T2 map reveals a decrease in the T2 values compared to the preoperative status. (**C**) Four months and (**D**) twelve months postoperatively, the cartilage surfaces seem to break up and the corresponding T2 values increase significantly.

**Figure 3:**
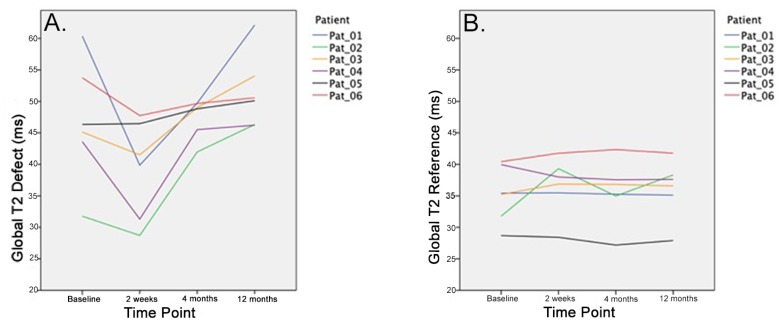
The global T2 values of the defect site decrease two weeks after treatment. However, an increase was observed in each patient after the first two postoperative weeks (**A**). No significant changes were observed at the reference site over the time course of 12 months (**B**).

**Figure 4:**
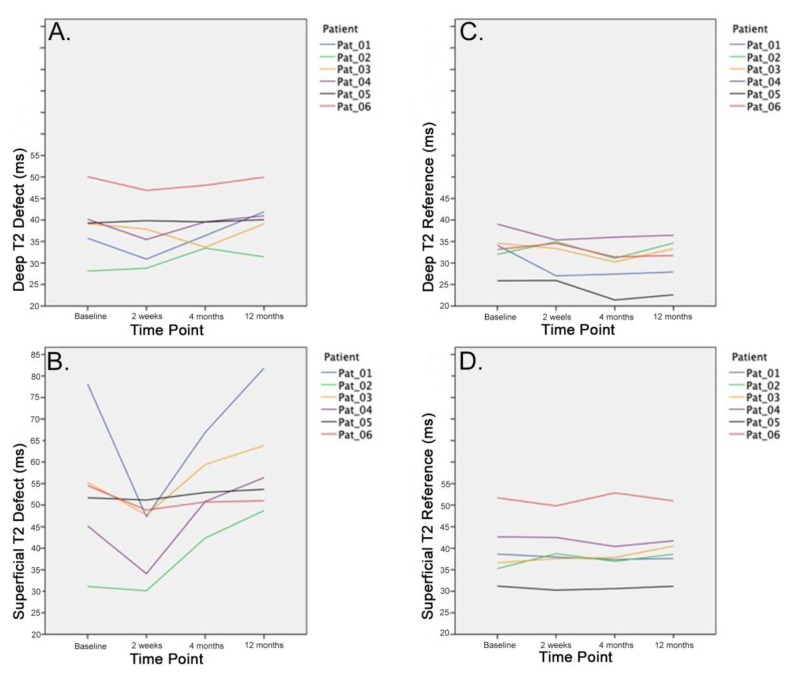
The mean T2 values of the deep (**A**) and superficial (**B**) layer of the defect and reference (**C**,**D**) site over the time course of 12 month are displayed for each patient. Radiofrequency chondroplasty leads to distinct increases in T2 values in the superficial layer.

The one-way ANOVA revealed a significant difference within global T2 values of the cartilage defect site for the different time points (*p* = 0.048). Further characterization by Duncan posthoc test found an initial trend for a decrease in cartilage global T2 values of the defect site from baseline to 2 weeks postoperatively (*p* = 0.071). However, the further follow-up assessment at 1 year revealed a significant difference with increasing global T2 values at the defect site compared to 2 weeks postoperatively (Duncan posthoc test < 0.05) (Table 1, Figure 2). No significant differences over time were observed for the global T2 values of the healthy reference site (Figure 3 and Figure 4).

### 3.3. Zonal T2 Values

Regarding the zonal assessment of T2 values after treatment, relevant changes were only found in the superficial layer, whereas the T2 relaxations times were more or less consistent over time in the deep layer (Table 1, Figure 4).

### 3.4. Morphological MRI

Morphological MRI underestimated the extent of cartilage damage potentially caused by radiofrequency chondroplasty. Despite a significant postoperative increase in T2 values, the morphological MRI cartilage assessment remained unchanged (ICRS grade 2) in four out of six knees. Cartilage deterioration was only seen in two knees on morphological MRI (Table 2).

## 4. Discussion

The present study evaluated the effect of radiofrequency chondroplasty for the surgical treatment of grade II patellar cartilage defects using high-resolution MRI and T2 mapping. Over the time course of 12 months, we were able to show that, 2 weeks after the treatment with radiofrequency chondroplasty, an initial normalization of T2 values compared to the healthy reference cartilage could be achieved. However, after 4 months and 12 months, the T2 values increased again and exceeded the preoperative values. The initial normalization of T2 values might be a result of a superficial “scar” or neo-surface formation [10,12,14], with less free mobile water protons within the treated cartilage tissue (Figure 2B). However, this neo-surface may be too stiff to be resistant against the shear stresses in the patellofemoral joint. As a consequence, it seems to break down over time and more and more free water protons infiltrate the cartilage tissue (Figure 2C,D). Zonal evaluation by deep and superficial layers shows that most changes occur within the superficial T2 values, whereas T2 values in the deep zone remain stable. This further supports the theory that degeneration starts in the superficial cartilage layer [24,28,36,39,40,41].

Based on the findings of this pilot study, radiofrequency chondroplasty used for the surgical treatment for ICRS grade II patellar cartilage defects may cause additional cartilage damage exceeding the preoperative condition. Despite the small sample size, this study provides important clinical information raising concerns about radiofrequency chondroplasty as a treatment option for grade II cartilage defects in the patellofemoral joint.

In the present study, quantitative MRI was used to monitor the effect of radiofrequency chondroplasty on grade II patellar cartilage defects. Quantitative MRI with T2 mapping was utilized because it has been shown to be an accurate and reliable method to monitor the condition of early (grade I/II) articular cartilage defects in order to avoid second-look arthroscopy [40,42]. Furthermore, Baum et al. showed that T2 relaxation times are a suitable, non-invasive tool to assess early cartilage degeneration in the knee joint [23]. For the purpose of this study, patellar cartilage was chosen because it is the thickest in the knee joint [43]. The thickness of patellar cartilage allows for division into a superficial and a deep layer for optimal T2 mapping [43].

On morphological sequences—even when performed with a high-resolution protocol—cartilage degeneration was observed in only two out of six cases. However, on quantitative MRI with T2 mapping cartilage deterioration in the superficial zone was observed in five out of six patients. This demonstrated the superior sensitivity of T2 mapping compared to morphological MRI in the detection of mild to moderate cartilage lesions (Table 2). Soellner et al. confirmed that quantitative MRI is more sensitive to detecting early cartilage degeneration [42].

Previous studies have analyzed the effect of radiofrequency chondroplasty for grade II–III cartilage defects based on a clinical score and radiographs. Owens et al. [12] reported on two-year results of patellofemoral cartilage defects treated with radiofrequency chondroplasty compared to mechanical debridement. The authors assessed the Fulkerson–Shea score preoperatively and 12 and 24 months postoperatively and showed superior clinical results in patients treated with radiofrequency compared to mechanical shaving. According to Owens et al., radiofrequency debridement is an effective tool to stabilize grade II–III cartilage defects. Spahn et al. [7] followed 47 patients who were treated either with bipolar temperature-controlled radiofrequency or mechanical debridement for 10 years. As in this study, the authors indicated surgery for meniscal pathologies and only targeted cartilage lesions as concomitant pathology. They reported a better clinical outcome and less progression of osteoarthritis for patients treated with radiofrequency chondroplasty. However, progression of osteoarthritic changes was only assessed on radiographs by measuring the joint space width.

Barber et al. [15] evaluated debridement versus debridement and additional radiofrequency chondroplasty for grade III defects by MRI. The authors assessed preoperative MRIs and MRIs 1 year postoperatively, looking for avascular necrosis but also edema and fluid changes within the bone. After a mean of 19 months postoperatively, the authors did not find any heat-related damage to the subchondral bone. However, the study did not report on any changes within the cartilage tissue. In line with Barber et al., the present study did not observe any pathological changes within the subchondral bone.

The above-mentioned studies evaluated the success of radiofrequency chondroplasty based on radiographs or morphological MRI although these methods are not precise enough to evaluate the status of cartilage on a biochemical level [28,36,41].

In the current study, patients were treated for a symptomatic meniscus tear and concomitant grade II patellar cartilage defects. One year after surgery, an increase in the KOOS and the UCLA score and a decrease in the VAS score was observed. Despite an improvement in clinical scores, the T2 values significantly increased, reflecting recurrent cartilage degeneration. None of the patients indicated patellofemoral problems before arthroscopy. The improvement in clinical scores can, therefore, be explained by the successful treatment of the meniscus lesion. Furthermore, it has been shown that early articular cartilage damages are not necessarily painful [18,44,45]. Concomitant grade II patellar cartilage lesions are frequently observed during arthroscopy [1]. Due to the lack of treatment options, radiofrequency chondroplasty appears to be a promising alternative in the management of these lesions, particularly since the radiofrequency probe is easy to use, giving the intraoperative impression of a long-lasting cartilage seal off. However, the present study suggests that caution is warranted, as radiofrequency chondroplasty may lead to additional cartilage damages exceeding the preoperative values.

The following limitations have to be acknowledged. First, the most important limitation of this study is the small number of patients. Initially, 20 patients were allocated for this study. However, assessing the first six patients four months out of radiofrequency chondroplasty, it was observed that T2 values were consistently elevated beyond preoperative levels in all patients. This study was, therefore, temporarily stopped until the 12 month MRI scans were available. The results at 12 months confirmed the T2 values at 4 months. This study was, therefore, terminated due to ethical considerations. Because of the small sample size, this study is underpowered to detect small effects. Second, in addition to radiofrequency chondroplasty, a medial meniscus trim was performed in all patients. Based on the clinical results, all patients benefited from arthroscopy. The meniscal pathology has to be considered as a confounder, and it therefore remains unclear whether the improvement in clinical scores is related to the meniscus trim or to the radiofrequency chondroplasty. Nevertheless, due to the fact that none of the patients reported preoperative symptoms associated with a patellofemoral pathology, the improvement in clinical scores is more likely related to the successful meniscus trim. Third, patients were mobilized full weight bearing on crutches for 2 weeks, which might have compromised the cartilage healing process. However, T2 values at the patellar cartilage defect site normalized with reference to healthy cartilage at 2 weeks, indicating that initial full weight bearing did not interfere with the formation of a neo-surface.

## 5. Conclusions

T2 mapping appears to be an ideal method to monitor cartilage degeneration after chondroplasty. Due to the observed postoperative cartilage damages on quantitative MRI, this study was terminated before completion. Based on the small sample size of this pilot study, radiofrequency chondroplasty seems to cause cartilage damage and may not have a long-lasting effect in the treatment of grade II patellar cartilage defects. Based on the findings of this study, we recommend only addressing the pathology which indicated arthroscopy and leaving concomitant cartilage lesions untreated.

## Figures and Tables

**Table 1:** Values of all measurements of the deep, superficial and global zone of the cartilage defect and the reference cartilage over the time course, beginning with the preoperative baseline measurement, followed by measurements at two weeks, four month and 12 months after treatment.

		Mean Values (ms)	Std.-Deviation	95% Confidence Level	
				Lower Border	Upper Border
Deep T2 Values Defect	**Baseline**	38.7	7.1	31.3	46.2
	2 Weeks	36.6	6.5	29.7	43.4
	4 Months	38.4	5.4	32.7	44.1
	1 Year	40.5	5.9	34.3	46.8
	*P*-value	0.756			
Superficial T2 Values Defect	Baseline	52.6	15.3	36.5	68.8
	2 Weeks	43.2	8.7	33.9	52.4
	4 Months	53.8	8.3	45.1	62.7
	1 Year	59.2	12.2	46.4	72.1
	*P*-value	0.146			
Global T2 Values Defect	Baseline	46.8	9.7	36.6	57.0
	2 Weeks	39.2	7.7	31.0	47.4
	4 Months	47.4	3.1	44.2	50.7
	1 Year	51.5	5.9	45.3	57.8
	*P*-value	0.048			
Deep T2 Values Reference	Baseline	33.1	4.3	28.6	37.6
	2 Weeks	31.8	4.2	27.4	36.3
	4 Months	29.6	4.8	24.5	34.7
	1 Year	31.1	5.1	25.8	36.4
	*P*-value	0.617			
Superficial T2 Values Reference	Baseline	39.3	7.1	31.8	46.8
	2 Weeks	39.5	6.4	32.7	46.2
	4 Months	39.4	7.3	31.6	47.1
	1 Year	40.1	6.4	33.3	46.9
	*P*-value	0.997			
Global T2 Values Reference	Baseline	35.2	4.5	30.4	40.0
	2 Weeks	36.6	4.5	31.8	41.4
	4 Months	35.6	4.9	30.5	40.8
	1 Year	36.2	4.6	31.3	41.0
	*P*-value	0.958			

**Table 2:** Preoperative and one-year postoperative grading of cartilage defects on morphological MRI according to the ICRS classification system, as well as absolute pre- and postoperative T2 values for each patient. Morphological MRI underestimates the degree of postoperative cartilage damage.

			Preoperative Values			Postoperative Values		
	ICRS Grade	T2 Deep	T2 sup.	T2 Global	ICRS Grade	T2 Deep	T2 sup.	T2 Global
Patient 1	2	35.76 ms	78.17 ms	60.37 ms	3	41.9 ms	81.85 ms	62.13 ms
Patient 2	2	28.12 ms	31.13 ms	31.77 ms	2	31.42 ms	48.71 ms	46.33 ms
Patient 3	2	39.13 ms	55.24 ms	45.13 ms	2	39.12 ms	63.83 ms	54.05 ms
Patient 4	2	40.17 ms	45.21 ms	43.61 ms	3	40.97 ms	56.39 ms	46.23 ms
Patient 5	2	25.87 ms	31.21 ms	46.35 ms	2	40.07 ms	53.66 ms	50.11 ms
Patient 6	2	50.06 ms	54.33 ms	53.75 ms	2	49.95 ms	50.97 ms	50.57 ms

sup.: superficial.
